# Penile Metastasis From Dedifferentiated Prostate Cancer With Low PSA Levels: A Case Report

**DOI:** 10.1002/ccr3.72863

**Published:** 2026-06-16

**Authors:** Xianqi Shen, Xinwen Nian, Bosi Zhang, Yan Wang, Zhenyang Dong, Min Qu, Xu Gao

**Affiliations:** ^1^ Department of Urology Changhai Hospital Shanghai China

**Keywords:** penectomy, penile metastasis, prostate cancer, prostate‐specific antigen

## Abstract

Penile metastasis from prostate cancer is rare and is associated with a poor prognosis; however, there is currently a lack of high‐level evidence to guide the management of this condition. This case report describes a 68‐year‐old male with prostate cancer who developed a penile metastasis during follow‐up. This occurred despite stable prostate‐specific antigen (PSA) levels after treatment with androgen deprivation therapy, combined with endocrine therapy and radiotherapy. Imaging studies and intraoperative frozen section pathology suggested a penile tumor. The patient subsequently underwent total penectomy combined with urinary diversion surgery. Postoperative histopathology confirmed poorly differentiated adenocarcinoma consistent with metastatic prostatic adenocarcinoma exhibiting morphological changes related to prior therapy. This case highlights the importance of maintaining clinical suspicion for penile metastasis in prostate cancer patients with stable PSA levels, especially in the context of tumor dedifferentiation, to facilitate early diagnosis and appropriate intervention.

## Key Clinical Message

This case serves as a clinical reminder that even in patients with stable PSA levels undergoing long‐term androgen deprivation therapy (ADT), clinicians must maintain a high index of suspicion for metastatic dedifferentiated prostate cancer when presenting with a penile mass of unknown etiology. Intraoperative frozen section biopsy is critical for determining the appropriate therapeutic strategy.

## 1 Introduction

Prostate cancer (PCa) is one of the most common malignancies in the male genitourinary system, with rapidly increasing incidence and mortality rates [1]. Advanced PCa typically metastasizes to bones or lymph nodes, posing significant challenges in clinical management [2]. Penile metastasis is exceptionally rare, occurring in approximately 0.3%–0.5% of cases [3]. The underlying mechanisms remain incompletely understood, and both diagnosis and treatment present considerable difficulties. As a critical component of the male reproductive system, involvement of the penis by metastatic lesion not only compromises physiological function but also significantly impairs quality of life. Furthermore, penile metastasis of PCa is generally associated with a poor prognosis, and lacks standardized treatment strategies supported by high‐level evidence [4]. Here, we present a patient of advanced PCa with penile metastasis and its related diagnosis and treatment, hoping to provide new insights for the clinical management.

## 2 Case History/Examination

A 68‐year‐old male was found to have an elevated prostate‐specific antigen (PSA) level (> 100 ng/mL) during a routine health examination in August 2021. Dilution testing revealed a total PSA of 368.17 ng/mL and a free PSA of 30.75 ng/mL. Multiparametric magnetic resonance imaging (mp‐MRI) of the prostate demonstrated an abnormal signal in the right peripheral zone, suggestive of prostate cancer. This abnormality involved the right seminal vesicle and multiple pelvic sites. A subsequent bone scan confirmed widespread skeletal metastases. On August 24, 2021, a transperineal systematic prostate biopsy guided by transrectal ultrasound (TRUS) was performed. Histopathological analysis confirmed the diagnosis of prostatic acinar adenocarcinoma with 7 of 12 biopsy cores positive and a Gleason score of 4 + 3 = 7.

On September 6, 2021, the patient began combined androgen deprivation therapy (ADT) and androgen receptor pathway inhibitor (ARPI), consisting of goserelin acetate (10.8 mg) and enzalutamide (160 mg, orally once daily). Throughout this treatment period until December 2024, the patient remained asymptomatic, with PSA levels well‐controlled and reaching a nadir of 0 ng/mL. However, a follow‐up contrast‐enhanced pelvic MRI performed on December 3, 2024, revealed an abnormal signal in the right prostatic gland with involvement of the right levator ani muscle and rectal wall (Figure 1A,B), despite a PSA level of 0.001 ng/mL.

**FIGURE 1:**
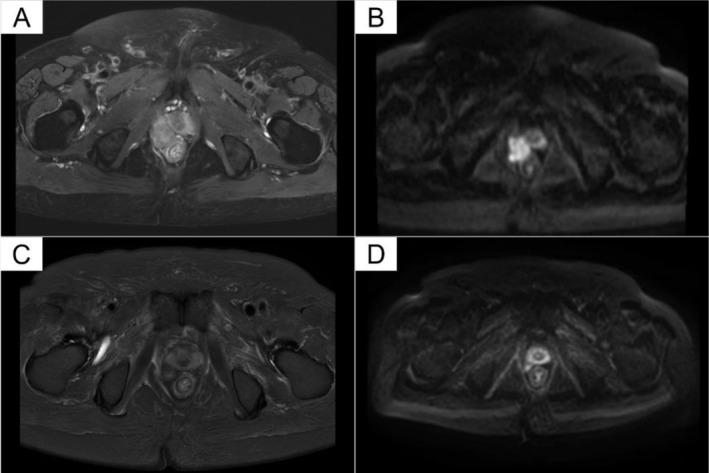
Contrast‐enhanced pelvic MRI revealed an abnormal signal in the right prostatic gland with involvement of the right levator ani muscle and rectal wall: (A) T2 before radiotherapy, (B) DWI before radiotherapy, (C) T2 after radiotherapy, and (D) DWI after radiotherapy. DWI: Diffusion weighted imaging.

Radiotherapy was initiated on December 10, 2024, targeting the prostate and seminal vesicles, (2.5 Gy × 28 fractions), and the pelvic region, (1.8 Gy × 28 fractions). Treatment concluded on January 17, 2025. After radiotherapy, PSA levels decreased to 0 ng/mL. Follow‐up MRI showed a reduction in the right peripheral zone lesion compared to the December 3, 2024 scan (Figure 1C,D). A bone scan indicated partial suppression of metabolic activity in previously documented skeletal metastases. Prostate‐specific membrane antigen positron emission tomography/computed tomography (PSMA PET/CT) suggested residual tumor activity in the right peripheral zone, a possible inflammatory focus in the left transitional zone, a metastatic left presacral lymph node, and residual active lesions in bones. PSA levels remained stable over the following months, with a nadir of 0.03 ng/mL. However, In June 2025, the patient reported a palpable solid nodule in the mid‐penis.

## 3 Differential Diagnosis, Investigations, and Treatment

The primary differential diagnosis included primary penile squamous cell carcinoma (SCC) and metastatic penile cancer. Given the patient's history of prostate cancer, despite the stable PSA levels, metastatic prostatic adenocarcinoma was considered a high‐priority diagnostic possibility. Penile ultrasound identified a solid nodule in the corpora cavernosa, raising suspicion for a neoplastic lesion. Subsequent contrast‐enhanced scrotal and testicular MRI revealed multiple nodules in the corpora cavernosa. These nodules exhibited isointense T1‐weighted and mildly hypointense T2‐weighted signals, with the largest measuring approximately 1.6 cm. Moreover, these nodules demonstrated diffusion restriction and ring enhancement on contrast imaging (Figure 2).

**FIGURE 2:**
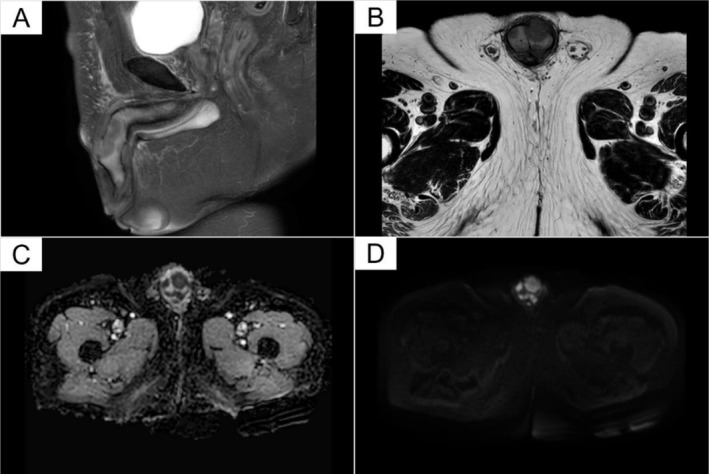
Contrast‐enhanced scrotal and testicular MRI revealed multiple nodules in the corpora cavernosa: (A) Sagittal of T2, (B) Axis of T2, (C) Axis of ADC, and (D) Axis of DWI. ADC: Apparent Diffusion Coefficient.

Considering the patient's symptoms of dysuria progressively worsened, to accurately determine the nature of the penile lesion and address the patient's symptomatic distress, we performed an intraoperative frozen section biopsy, and the pathology indicated a poorly differentiated malignant tumor in the corpus cavernosum. Given that the lesion was located close to the penile root with extensive involvement of the corpora cavernosa, radical resection was required to ensure adequate oncological control, and no sufficient functional penile length could be preserved. After thorough intraoperative communication and informed consent with the patient's family, total penectomy with perineal urethrostomy was performed. Final histopathological examination revealed a 3 × 2.5 × 2 cm tumor in the penile shaft. Microscopically, the tumor cells exhibited large size, hyperchromatic nuclei, significant atypia, and were arranged in irregular glandular and nested patterns with central comedonecrosis. Immunohistochemical staining results were as follows: AE1/AE3 (+), CAM5.2 (+), EMA (+), ERG (−), P504S (focal +), NKX3.1 (−), CK5/6 (rare +), p40 (rare +), p63 (rare +), PSMA (rare +), AR (−), PTEN (−), NSE (−), c‐Myc (−), SSTR2 (−), CgA (−), Syn (rare +), CD56 (focal +), INSM1 (−), BRCA‐1 (+), p53 (60%+), Ki‐67 (60%+), PSA (−). The pathological diagnosis was poorly differentiated adenocarcinoma of the corpus cavernosum. Based on morphological and immunohistochemical features, the findings were consistent with metastatic prostatic acinar adenocarcinoma with treatment‐related changes (Figure 3).

**FIGURE 3:**
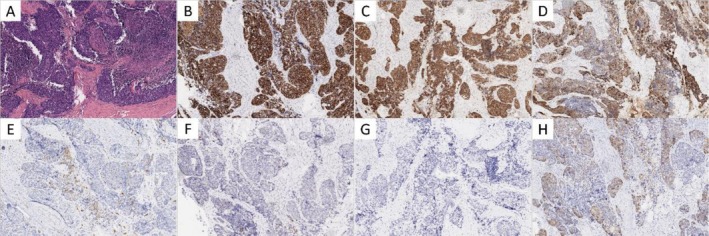
Pathological and immunohistochemical findings of the metastatic penile tumor: (A) Pathological and immunohistochemical staining, (B) Anti‐Cytokeratin Antibody Cocktail AE1/AE3 (AE1/AE3) (+), (C) Cellular Adhesion Molecule 5.2 (CAM5.2) (+), (D) alpha‐methylacyl‐CoA racemase (P504S) (focal +), (E) prostate‐specific membrane antigen (PSMA) (rare +), (F) Protein 63 (P63) (rare +), (G) Protein 40 (P40) (rare +), and (H) Cluster of Differentiation 56 (CD56) (focal +).

## 4 Discussion

Prostate cancer has become one of the most common malignancies among males worldwide [1]. In China, approximately 60% of prostate cancer patients are diagnosed with distant metastasis at initial presentation [5]. Penile metastasis, though rare (occurring in 0.3%–0.5% of cases), is associated with poor oncological outcomes and often signifies advanced disease [3, 4]. However, current clinical literature on penile metastasis from prostate cancer consists largely of case reports, with a limited number of systematic reviews. This gap contributes to limited clinical awareness and a tendency to misdiagnose these lesions as benign conditions, including Peyronie's disease [6]. Therefore, a thorough analysis of the clinical features, diagnostic approaches, and treatment strategies for penile metastasis is essential to improve early detection and treatment precision.

The clinical manifestations of penile metastasis are nonspecific and may include palpable nodules, pain, urinary symptoms, and erectile dysfunction, often leading to diagnostic confusion [7]. For instance, Ogawa et al. reported a 77‐year‐old patient presenting with urinary frequency, nocturia, and a painless penile nodule, ultimately diagnosed as metastatic prostate adenocarcinoma with concurrent bone and lymph node involvement [8]. Similarly, Cante et al. described a case of mucinous adenocarcinoma with penile metastasis where the patient presented with penile pain and experienced significant symptom relief following radiotherapy combined with endocrine therapy [9]. Yu Dai emphasized that penile metastasis can mimic Peyronie's disease, underscoring the importance of considering malignancy in cases of penile induration [6]. Pathologically, most penile metastases originate from prostatic adenocarcinomas; however, some cases exhibit neuroendocrine differentiation, which is associated with greater aggressiveness and treatment resistance [10]. In the present case, penile metastasis emerged during endocrine therapy despite well‐controlled serum PSA levels. Histopathological assessment revealed a poorly differentiated adenocarcinoma, suggesting a potentially aggressive subtype. The mechanisms underlying penile metastasis remain incompletely elucidated. Notably, its occurrence often coincides with metastases to other sites, reflecting rapid disease progression and an unfavorable prognosis.

The diagnosis of penile metastases relies on clinical presentation, imaging studies, and pathological biopsy. However, because clinical symptoms are nonspecific and often mimic other penile pathologies, imaging plays a critical role in differential diagnosis. Ultrasonography may detect penile masses and abnormal vascularity, but it has limited specificity. Functional imaging such as positron emission tomography/computed tomography (PET/CT) is widely utilized for detecting metastatic prostate cancer. Bianchi et al. demonstrated the utility of 18F‐fluorocholine PET/CT in identifying penile metastases, highlighting its advantage in detecting rare metastatic sites such as the penis at an early stage [11]. Thus, a comprehensive diagnostic approach integrating imaging and histopathology is crucial. Nevertheless, histopathological examination remains the gold standard for definitive diagnosis. As reported by Yu Dai et al., a case initially misdiagnosed as Peyronie's disease due to penile induration was ultimately confirmed as metastatic disease through biopsy, underscoring the indispensable role of pathological verification [6]. Moreover, the selection of staining markers for immunohistochemistry is of paramount importance, as it exerts a direct influence on the accuracy of disease diagnosis [12].

Treatment strategies for penile metastasis primarily focus on symptom relief and prolongation of survival, as no standardized therapeutic protocol exists. Endocrine therapy serves as the cornerstone of systemic treatment for prostate cancer and is commonly employed in cases with penile involvement. Previous studies have reported that combining endocrine therapy with radiotherapy or chemotherapy can alleviate symptoms and improve quality of life. For instance, Ogawa et al. documented a case of advanced disease where a combination chemotherapy regimen including estramustine, paclitaxel, and carboplatin resulted in tumor regression and decreased tumor markers; this suggests potential efficacy in carefully selected patients [8]. Surgical intervention is generally reserved for diagnostic purposes or symptomatic relief [9, 11]. In the present case, despite well‐controlled tumor markers following radiotherapy and androgen deprivation therapy (ADT), which is a form of endocrine therapy, the penile lesion progressively enlarged, leading to obstructive urinary symptoms. Consequently, after thorough discussion and informed consent from the patient and family, total penectomy was performed.

Current understanding of penile metastasis from prostate cancer is primarily derived from isolated case reports, with a notable absence of large‐scale, multi‐center systematic studies. This limitation hinders comprehensive insights into its underlying pathogenesis, optimal diagnostic approaches, and treatment strategies. Evaluations of therapeutic efficacy vary across studies: some reports advocate for combined chemotherapy and endocrine therapy, while others emphasize the role of radiotherapy in symptom control. The lack of long‐term follow‐up data further constrains a holistic assessment of treatment outcomes and prognosis.

In summary, penile metastasis from prostate cancer represents a rare and prognostically unfavorable manifestation of advanced disease. Its heterogeneous clinical presentation often leads to misdiagnosis. Imaging studies—particularly PSMA PET/CT—and histopathological examination are essential for accurate diagnosis; moreover, combining multiple diagnostic modalities is crucial for comprehensive evaluation. Treatment options—including endocrine therapy, radiotherapy, chemotherapy, and surgery—should be tailored to the patient's clinical condition. Future research should prioritize multicenter collaborations to establish large clinical databases. Additionally, efforts should focus on elucidating the molecular mechanisms and biomarkers of penile metastasis, as well as developing precision diagnostic tools and personalized treatment strategies.

## 5 Conclusions

The diagnosis of penile metastasis from prostate cancer poses significant diagnostic challenges due to its extreme rarity and nonspecific presentation. A comprehensive evaluation—combining physical examination, imaging techniques, and pathological assessment—is crucial for a definitive diagnosis. Consequently, management should be guided by a personalized strategy tailored to the individual's clinical manifestations.

## Author Contributions

**Xianqi Shen:** conceptualization, data curation, formal analysis, funding acquisition, writing – original draft, writing – review and editing. **Xinwen Nian:** funding acquisition, visualization. **Bosi Zhang:** conceptualization, data curation, writing – original draft. **Yan Wang:** data curation, investigation, visualization. **Zhenyang Dong:** data curation, writing – review and editing. **Min Qu:** conceptualization, data curation, formal analysis, writing – review and editing. **Xu Gao:** conceptualization, data curation, funding acquisition, writing – original draft, writing – review and editing.

## Funding

This work was supported by Shanghai Hospital Development Center (SHDC2025CCS007), Shanghai's 2024 “Science and Technology Innovation Action Plan” Launch Star Project (24YF2758900), and 2025 Basic Medical Research Youth Training Camp Special Project (2025QN015).

## Ethics Statement

The experiments were approved by the ethics committee of Shanghai Changhai Hospital.

## Consent

Written informed consent was obtained from the patient to publish this case report in accordance with the journal's patient consent policy.

## Conflicts of Interest

The authors declare no conflicts of interest.

## Data Availability

The data that support the findings of this study are available from the corresponding author upon reasonable request.
